# Design and Modeling of a Microfluidic Coral Polyps Culture Chip with Concentration and Temperature Gradients

**DOI:** 10.3390/mi13060832

**Published:** 2022-05-26

**Authors:** Shizheng Zhou, Edgar S. Fu, Bingbing Chen, Hong Yan

**Affiliations:** 1 State Key Laboratory of Marine Resource Utilization in South China Sea, Hainan University, Haikou 570228, China; xpzzzsz@hotmail.com (S.Z.); ice.square@yahoo.com (B.C.); 2 School of Computing and Information, University of Pittsburgh, Pittsburgh, PA 15260, USA; esf27@pitt.edu

**Keywords:** coral polyps, multivariable, microfluidic chip, temperature gradient, concentration gradient, numerical simulation

## Abstract

Traditional methods of cultivating polyps are costly and time-consuming. Microfluidic chip technology makes it possible to study coral polyps at the single-cell level, but most chips can only be analyzed for a single environmental variable. In this work, we addressed these issues by designing a microfluidic coral polyp culture chip with a multi-physical field for multivariable analyses and verifying the feasibility of the chip through numerical simulation. This chip used multiple serpentine structures to generate the concentration gradient and used a circuit to form the Joule effect for the temperature gradient. It could generate different temperature gradients at different voltages for studying the growth of polyps in different solutes or at different temperatures. The simulation of flow field and temperature showed that the solute and heat could be transferred evenly and efficiently in the chambers, and that the temperature of the chamber remained unchanged after 24 h of continuous heating. The thermal expansion of the microfluidic chip was low at the optimal culture temperature of coral polyps, which proves the feasibility of the use of the multivariable microfluidic model for polyp culture and provides a theoretical basis for the actual chip processing.

## 1. Introduction

**Figure 1:**
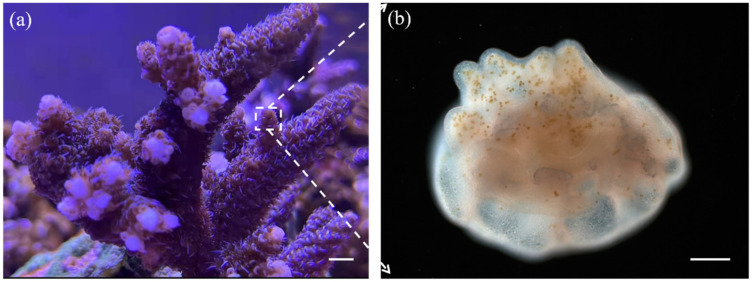
Coral and coral polyps. (**a**) Coral is composed of coral reefs, coral polyps and symbiosis microorganisms. (scale bar = 1 cm). (**b**) Polyps and symbiotic microorganisms; the yellow dots refer to *Symbiodinium* (scale bar = 1 mm).

Coral reef ecosystems are rich in biodiversity and biomass and have an extremely high primary productivity. However, increasing temperatures, the degradation of the marine environment, and human activities have resulted in the occurrence of coral reef bleaching events on a large scale. Coral is a biological structure composed of coral reefs, coral polyps and microorganisms that live in symbiosis with the polyps. Coral polyps have a large number of symbiotic microorganisms attached to their bodies and surfaces [1], such as *Symbiodinium*, as shown in Figure 1. Coral polyps and their symbiotic microorganisms are associated with stringent environmental requirements. Changes in pH [2], temperature [3], light intensity [4], and ionic concentration [5] will affect their symbiotic relationship and coral metabolism, but the exact symbiotic mechanism involved is not known at present [6]. Currently, in the laboratory, coral polyps are mainly cultured at temperatures of 24 ± 2 °C and pH levels of 8.1 ± 0.2 [7,8], but at elevated temperatures (e.g., 32 °C) they undergo bleaching [8,9]. The investigation of the response mechanisms of corals under environmental stress cannot be made comprehensive enough by studying coral communities alone. The investigation of biological processes occurring at the tissue or cellular level in individual coral polyps and the acquisition of information on the growth and physiology of individual coral polyps under different environmental variables are necessary in order to study the metabolic mechanisms of corals. Currently, coral polyps are mainly cultured using Petri dishes with solid media [10], which is an inefficient method for exploring multiple coral polyps’ growth variables. Moreover, the Petri dish cannot provide a dynamic growth environment for coral polyps and is susceptible to more external disturbances. In recent years, advances in microfluidics and molecular techniques have made it possible to study the coral polyp at the single-cell level. Observation, detection, and analysis by microfluidics provide particularly powerful tools for the study of the physiological ecology and biological behavior of the coral polyp [11,12,13,14,15,16].

A microfluidic chip is a device that uses micrometer-scaled fluidic channels to handle fluids, and it provides a convenient platform for cell culture and tissue culture [17]. The scale of microchannels matches the size of the cells, which facilitates analytical studies at the single-cell level [18]. Microfluidic chips can be used to flexibly design cell culture chambers to form a relatively independent and closed three-dimensional environment; to precisely control culture conditions, such as the solution concentration [19,20] and temperature [21] within the chamber; and to simulate the real living environment of cells through some elaborate microstructures [22]. However, most microfluidic cell culture chips have a single function and can only simulate and analyze one environmental parameter at present.

The numerical simulation method has been widely used in the research and development of microfluidic chips [23]. By solving the governing equations of fluid dynamics, the flow field, solution concentration distribution, and particle trajectory within the microchannel can be simulated to optimize the chip structure [24,25]. By solving the thermodynamic equations to understand the temperature change of the microfluidic chip under electrothermal conditions, the experimental cost of chip fabrication can be reduced [26,27,28]. The deformation of the chip under different conditions can also be understood by finite element simulation [29].

In this study, we designed and modeled a microfluidic chip for multi-parameter coral polyps culturing. We performed numerical simulations on the generation of the concentration, the temperature change at different voltages, and the thermal expansion of the chip to verify the chip’s feasibility. The results showed that the chip could stably form a concentration gradient due to its serpentine structure. In addition, by changing the voltage value, the chip could stably form a variety of temperature gradients for a long time, which could be used to explore the growth and physiological conditions of coral polyps under different heat stresses. The microfluidic chip model can provide a new platform for the ecological research of coral reefs in the future for analyzing the growth and physiological processes of coral polyps under different environmental pressures, as well as providing a reference for the research on other marine symbiotic micro-ecosystems.

## 2. Materials and Methods

### 2.1. Design and Modeling of Microfluidic Chip

**Figure 2:**
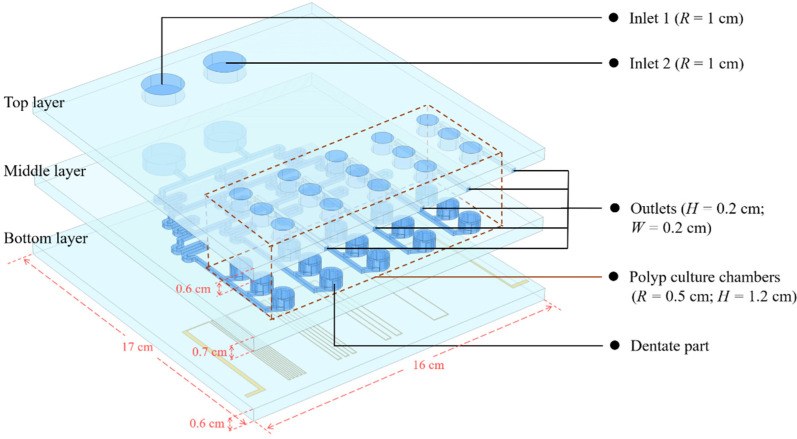
The structure diagram of the microfluidic polyp culture chip.

We constructed a three-dimensional model of a microfluidic chip for coral polyp culturing, coupled with dilute matter transfer, electromagnetic heat, non-isothermal flow, and thermal expansion physical fields to simulate the generation of concentration gradients in microchannels, temperature conduction between solids and fluids, and the deformation of solids under the action of electromagnetic heat. The three-dimensional geometric model is shown in Figure 2, where *R* represents the radius of the circular channel, *H* represents the height, and *W* represents the width of the channel.

The chip is divided into three layers, where the top layer is the inlet and outlet layer, the middle layer is the concentration gradient generation layer, and the bottom layer is the circuit layer. The material of the chip is glass (silica glass). The height of top layer is 0.6 cm, and the solution is injected through two circular holes with a radius of 2 cm. The outlet channels are 2 mm wide and 2 mm high, and each outlet channel collects metabolic waste from three individual coral polyp culture chambers. The middle layer can mix the solution sufficiently and form a concentration gradient through a multiple serpentine structure of the layer—for example, through the injection of solutions with different pH levels to form an acid–base gradient or through the injection of nutrients and buffers to form a substance concentration gradient. The five columns of gradients formed by the serpentine microchannels are numbered from left to right as A, B, C, D and E. The solution of each column flows to three independent coral polyp culture chambers. The three chambers are numbered sequentially as 1, 2 and 3, so each polyp culturing chamber can be named by the combination of the column number and the chamber number; for example, A1 indicates the 1st chamber under the gradient of column A. Each culture chamber is assembled with a dentate part, and we hope to use this part to fix the coral polyps, change the flow field, and improve the efficiency of mass transfer and heat transfer of the solution in the chamber. The solution gradually fills the chamber until the height of the solution reaches the outlet of the channel, which makes the coral polyps able to be in full contact with the solution for better growth and metabolism. The bottom layer is the circuit layer, and the thickness of the circuit in the layer is 10 µm. Different circuit patterns result in different levels of Joule heat and transfer to the chip and polyp culture chambers. The temperature of the fluid in the different culture chambers can be controlled by the reasonable design of the circuit pattern on the bottom layer.

### 2.2. Governing Equations

#### 2.2.1. Laminar Flow

The fluid within the microchannel of the chip follows the mass, momentum, and energy conservation equations. In the chip, the characteristic scale of fluid flow is mainly in the micrometer range, which is much larger than the average free range of molecules, meaning that the fluid flow can be regarded as continuous and any point in the flow field will satisfy the mass conservation equation (Equation (1)). In this coral polyps culture chip, the accumulated rate of the momentum of the microfluid is equal to the sum of the external forces acting on it, which satisfies the momentum conservation equation (Equation (2)); for the convenience of calculation, the heat transfer within the chip only considers the solid and the fluid and ignores the heat generation due to viscous dissipation. Assuming that the fluid is a Newtonian fluid, the energy conservation equation can be rewritten as the temperature equation (Equation (3)). The equation is as follows:

(1) $$\frac{\partial \rho }{\partial t}+\nabla \left(\rho \cdot u\right)=0$$

(2) $$\rho \left(\frac{\partial u}{\partial t}+\left(u\cdot \nabla \right)u\right)=\nabla \left\{-pI+\mu \left[\nabla u+{\left(\nabla u\right)}^{T}\right]\right\}+F$$

(3) $$\rho C_{p}\frac{\partial T}{\partial t}+\rho C_{p}u\cdot \nabla T+\nabla \cdot \left(-k\nabla T\right)=Q_{e}$$

where ρ is the fluid density; u is the velocity vector of the fluid; t is the time of fluid flow; I is the unit vector; μ is the viscosity coefficient of the fluid; F is the stress per unit mass of fluid; $C_{p}$ is the specific heat capacity; T is the temperature; k is the thermal conductivity; and $Q_{e}$ is the Joule heat generated by the circuit in the circuit layer, which is the only heat source in the chip.

#### 2.2.2. Solution Diffusion and Mixing

The coralline culture chip uses a multi-layered serpentine microchannel structure. In the simulation, the solute diffusion equilibrium equation is solved by coupling the dilute matter transfer physical-field interface to obtain the solution concentration distribution in the microchannel. Since there is no chemical reaction between the solutions, the solute diffusion equilibrium equation is as follows:

(4) $$\frac{\partial c}{\partial t}+\nabla \cdot \left(-D\nabla c\right)+u\cdot \nabla c=0$$

where c is the concentration; D is the diffusion coefficient; and u is the velocity vector of the fluid.

#### 2.2.3. Joule Heat

Applying different magnitudes of voltage at the two ends of the circuit in the bottom layer to make it be under a uniform electric field generates Joule heat. The Joule heat generated by the circuit at this time is calculated using the following equation:

(5) $$Q_{e}=J\cdot \frac{U}{d}$$

where $Q_{e}$ is the Joule heat generated by the circuit; J is the current density; U is the potential difference between the two ends of the circuit; and d is the distance between the two ends of the circuit.

The Joule heat generated by the circuit, as the only heat source, is conducted to the chip glass in the steady state, and the glass structure is cooled by a natural heat exchange with the outside air as well as convective heat exchange with the fluid; the heat transfer equation is shown in Equation (3). Due to the different thermal expansion coefficients of the materials, the rise in temperature also causes thermal stresses leading to the deformation of the chip structure. In this study, the degree of thermal expansion of the chip is determined by the von Mises effective stress and the displacement is due to thermal deformation.

### 2.3. Parameter Setting

Five physical fields were used in the simulation: laminar flow, current, solid and fluid heat transfer, thermal expansion, and dilute matter transfer. Among these, the laminar flow and dilute matter transfer physical fields determined the fluid flow velocity and the concentration gradient distribution, and the change in fluid flow velocity affected the temperature in the subsequent polyp culture chamber; the current and solid and fluid heat transfer determined the magnitude and distribution of Joule heat, and the deformation of the microfluidic chip under Joule heat could be calculated by the thermal expansion physical field. In this study, the designed microfluidic chip was to be simulated in the following steps after establishing the three-dimensional geometric model and governing equations of the microfluidic chip.

#### 2.3.1. Material Property Setting

The material of the fluid in the microchannel was set to water, the circuit material in the circuit layer was set to copper, and the rest of the material was set to silica glass. The specific material properties are shown in Table 1.

#### 2.3.2. Boundary Condition Setting

The initial flow rate of both inlet 1 and inlet 2 were set to 0.01 mL/min, the concentrations were 0.1 mol/m^3^ and 0.5 mol/m^3^, and the diffusion coefficient of the fluid was 10^−9^ m^2^/s. The voltage of the cathode of the circuit was set to 0 V, and the voltage of the anode was set to 1 V, 2 V, 3 V, 4 V, and 5 V, respectively, to investigate the heat transfer of the chip under different voltages. Natural thermal convection would occur between the outer surface of the chip and the air (at a temperature of 20 °C), and the air heat transfer coefficient was 5 W/(m^2^·K). The heat transfer coefficient of the fluid inside the microchannel changed with the temperature.

#### 2.3.3. Solver Setting

Due to the large number of physical fields that needed to be coupled, in this study a distributed solver method was used. Firstly, the physical fields of laminar flow and dilute matter transfer were solved to obtain the flow distribution in the microchannel under isothermal conditions. Then, we coupled the current physical field and solid and fluid heat-transfer physical field to obtain the Joule heat generated by the circuit and the approximate temperature distribution on the chip. Further, the above two results were used as the initial values for solving the non-isothermal flow to calculate the concentration gradient distribution and temperature distribution of the microfluidic chip. The temperature changes with time in the culture chamber were simulated under transient conditions. Finally, we couple all physical fields in the steady-state and calculated the thermal deformation of the overall chip structure to evaluate the feasibility of the chip under heat.

## 3. Results and Discussion

### 3.1. Flow Field Simulation

**Figure 3:**
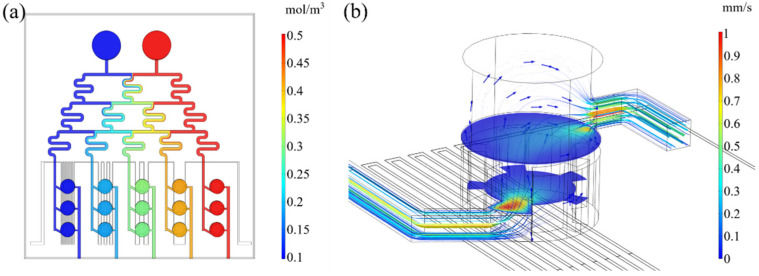
Flow field distribution diagram in the microchannel. (**a**) Concentration distribution map in microchannel under steady state. The values of the concentrations are shown in the legend on the right. (**b**) Streamline distribution of the flow field in a culture chamber. The values of the flow rate are shown in the legend on the right, and the shear rates are represented in terms of the thickness of the flow lines.

The simulation of the concentration distribution of the chip flow field at steady state is shown in Figure 3a. The concentration of the solution flowing in inlet 1 (blue) was 0.1 mol/m^3^, and in inlet 2 (red) it was 0.5 mol/m^3^. After mixing three times, the concentration distributions in the serpentine channel of columns A to E were 0.1 mol/m^3^, 0.2 mol/m^3^, 0.3 mol/m^3^, 0.4 mol/m^3^, and 0.5 mol/m^3^, respectively. The solutions of different concentrations were mixed efficiently in the serpentine channel and they formed a stable concentration gradient distribution by the time they reached the culture chamber.

Under steady-state, the distribution of the flow field in the culture chamber is shown in Figure 3b (taking the A1 chamber as an example), represented as streamlines. When the fluid flows into the chamber, due to the dentate part, a turbulent flow formed locally in the chamber, enabling the solute to be effectively transferred to all parts of the chamber, prolonging the time of the fluid on the heat source.

A series of point probes were set at the inlets of the polyp culture chambers to measure the flow rates of the solution as it flowed into each chamber, and domain probes were set inside the chambers to measure the average flow rate at steady state. The results are shown in Table 2. It can be seen that the flow velocity distribution of the flow field between columns of the chip was more uniform, and the average flow velocity in the chamber channel was about 0.34 mm/s. Since the unit volume of the chamber was much larger than the unit volume of the microchannel, the flow velocity of the solution gradually became slower when entering the chamber.

### 3.2. Temperature Field

When the constant current passed through the circuit, the heat generated by the Joule effect, as the only heat source on the microfluidic chip, formed a heat balance inside the chip through solid and fluid transfer, air cooling, and thermal radiation. The degree of temperature variation was related to the heat transfer method and the properties of the materials applied in the region, such as thermal conductivity and specific heat capacity.

**Figure 4:**
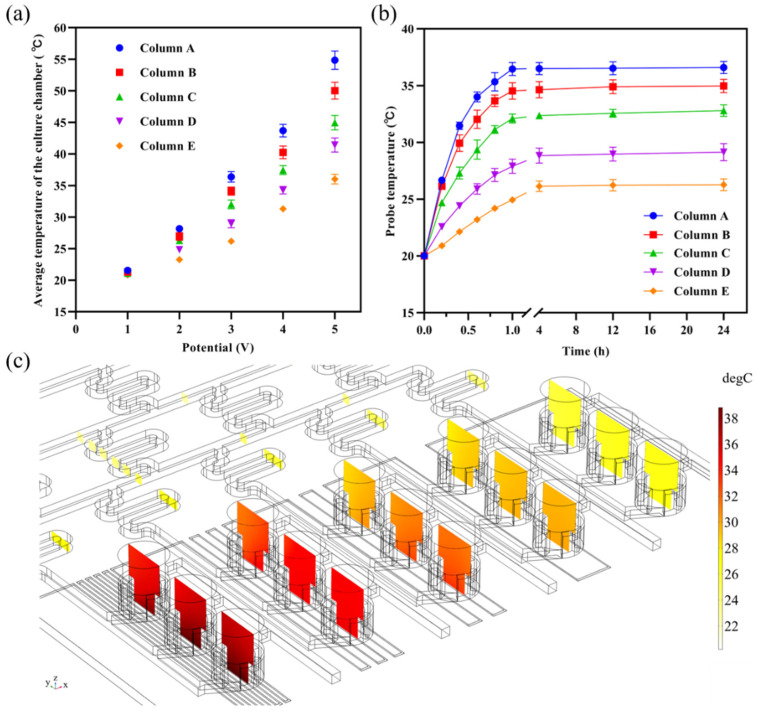
Temperature profiles of the coral polyp culture chip. (**a**) The average temperature of polyp culture chambers under different potentials. (**b**) Temperature variation over time within the coral polyp culture chambers. (**c**) Temperature profile of polyp culture chip at 3 V voltage under steady state. The values of the temperature are shown in the legend on the right.

The magnitude and distribution of temperature in the coral polyp culture chip at different voltages were coupled with a laminar flow, current, solid and fluid heat transfer, and dilute matter transfer physical fields. The average temperatures inside the culture chambers at 1 V, 2 V, 3 V, 4 V, and 5 V are shown in Figure 4a. At 1 V, the average temperature of the 5 columns of chambers was relatively uniform and closed to the external temperature (20 °C), while the average temperature gradually differed in the potential increase. The optimum growth temperature range for coral polyps in the field environment was from 22 °C to 28 °C. When the electric potential was 2 V, the temperature range of coral polyp culture chambers was between 23 °C and 28 °C, and the temperature difference between each chamber was 1.0 ± 0.2 °C, which were all within the optimum growth temperature range. Thus, the growth metabolism of coral polyps under different solutions could be investigated by injecting solutions with different concentrations to form a concentration gradient, or by injecting solutions with different pH levels to form a pH gradient at a 2 V potential. The outlets of each column of chambers were independent of each other and the metabolites of the coral polyps could be collected for downstream metabolic analysis. When the potential was 4 V or 5 V, the temperatures inside the chambers were too high and would directly lead to the death of the coral polyps. In contrast, when the electric potential was 3 V, the temperature range of coral polyp culture chambers was between 26 °C and 36 °C, which was an optimal temperature condition for studying coral bleaching and exploring the tolerance of coral polyps under unsuitable and gradually increasing environmental temperatures. This facilitated the selection of coral polyps tissues with high temperature tolerances, which was important for the prevention and management of coral bleaching.

The variation in temperature within each chamber with time at 3V is shown in Figure 4b, with an initial temperature of 20 °C for the chip. Due to the relatively slow flow of fluids within the chamber and the dentate part that extended the residence time of the fluid at the heat source, the heat was able to be transferred slowly and uniformly throughout the solution. The temperature inside the chamber remained stable after 24 h of continuous heating, which is a guaranteed condition for the long-term cultivation of coral polyps in the chamber.

The temperature cross-section map inside the coral polyp culture chambers at 3 V is shown in Figure 4c. For the whole chip, the highest temperature region was located in the lower-left part of the circuit, which included multiple U-shaped structures of the circuit; the lowest temperature region was located at the inlet of the chip. Because the material of the solid part in the chip was silica glass, the thermal conductivity was 1.38 W/(m^2^·K), while the thermal conductivity of the fluid (water) in the microchannel was between 0.59 W/(m^2^·K) and 0.62 W/(m^2^·K) with a temperature change at atmospheric pressures. Due to the higher specific heat capacity of water, it required more heat to be absorbed than the solid material, so the temperature of microchannel in the middle layer was lower than that of the surrounding solid domain, and the change in temperature in the microchannel mainly occurred in the coral polyp culture chambers.

The temperature of the coral polyp culture chamber decreased gradually from left to right; the lighter the color of the section was, the lower the temperature was. In the cross-sectional map, the lowest temperature of the fluid was found at the chip inlet. As the solution flowed into the chamber, the solution within the chamber absorbed the heat transferred from the surrounding solid parts and the bottom circuit, which was reflected in the form of increasing temperatures. The temperature of the chambers under different concentration columns decreased column by column, and the temperatures in each chamber were more uniform. The probes were set up to determine the average temperature of the five chamber columns, which were 36.4 °C, 34.3 °C, 32.3 °C, 30.0 °C, and 26.3 °C for columns A–E, respectively.

### 3.3. Thermal Deformation

**Figure 5:**
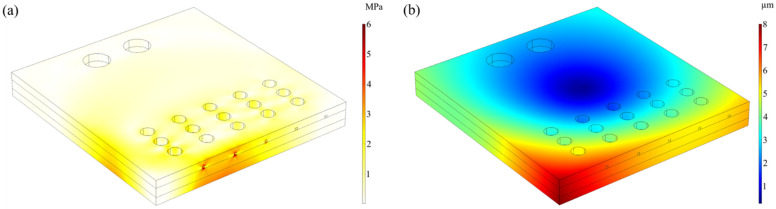
Thermal stress on microfluidic chips. (**a**) Von Mises effective stress caused by heat. The values of von Mises effective stress are shown in the legend on the right. (**b**) Displacement caused by heat. The values of displacement are shown in the legend on the right.

The thermal deformation of the chip at this potential was simulated at 3 V to evaluate the feasibility of the use of the chip under realistic conditions. As shown in Figure 5a, the maximum effective stress due to Joule heating was about 6 MPa, which occurred at the copper circuit below the coral polyp culture chamber in column A. The yield stress of silica glass was roughly 250 MPa, which meant that the components of the microfluidic chip remained structurally intact under the simulated heating capacity.

The total displacement of the chip due to Joule heat is shown in Figure 5b. The chip produced the maximum displacement due to heat at the lower-left corner, which produced a displacement of 8 µm. At the 3 V potential, the average displacement of the chip was about 4.6 µm, which was much smaller than the width and depth of the microchannel in the chip and the diameter of the coral polyp culture chamber, so it can be assumed that the displacement due to Joule heat did not affect the experimental results.

## 4. Conclusions

In this study, we designed a multivariate analytical coral polyp culture microfluidic chip that can form concentration gradients and temperature gradients, and every coral polyp culture chamber within the chip was independent of one another. The simulation of the flow field of the chip showed that the flow–velocity distribution between columns was relatively uniform, and the concentration gradient could be formed by mixing two different concentrations of solutions stably through the serpentine structure. The simulation of the chip temperature field showed that the chip could form a temperature gradient across the columns of the coral polyp culture chambers by changing the magnitude of the electric potential. At a potential of 2 V, the temperature range of the culture chambers was within the optimal range for the coral polyps, allowing the investigation of the growth and metabolism of individual coral polyp under different concentrations of solutions. At a potential of 3 V, the chambers were at ideal temperature conditions for coral bleaching, allowing the exploration of the tolerance of coral polyps under different environmental temperatures. Under the condition of applying a 3 V voltage for a long time, the temperatures of the polyp culture chambers could also be kept constant, and no noticeable thermal deformation found in the chip. The dentate structure in the chamber could make the solution form a forced turbulence, solve the dead zone and mass transfer problems in the culture chamber, increase the contact time between the heat source and the fluid, and make the heat transfer in the chamber stable and sufficient.

The chip is also relatively simple to produce, consisting of three layers of silica glass and copper electrodes. In the fabrication of the chip, the top and middle microfluidic channels and the circuit pattern of the bottom layer can be engraved by laser cutters, and the residual debris in the microchannels can be cleaned by an ultrasonic cleaning machine. After that, the entire chip is fabricated by a direct hot-press bonding process. In the future, we will conduct on-chip coral culture experiments through this microfluidic platform with the ability to analyze multiple environmental parameters to better understand the effects of the culture environment on the growth process of coral polyps and coral symbiotic algae at the cellular and tissue level.

## Figures and Tables

**Table 1:** Material properties.

Material Property	Copper	Silica Glass
Electrical conductivity (S/m)	6.0 × 10^7^	1.0 × 10^−14^
Constant-pressure heat capacity (J/(kg·K))	385	703
Relative dielectric constant	1	3.75
Density (kg/m^3^)	8960	2203
Thermal conductivity (W/(m^2^·K))	400	1.38
Resistivity (Ω·m)	1.7 × 10^−8^	-

**Table 2:** The flow velocity of each polyp culture chamber.

Column	Concentration (mol/m^3^)	Inlet Flow Velocity (mm/s)	Average Flow Velocity (mm/s)
A	0.1	0.56 ± 0.04	0.33 ± 0.01
B	0.2	0.60 ± 0.05	0.34 ± 0.01
C	0.3	0.58 ± 0.07	0.34 ± 0.01
D	0.4	0.57 ± 0.08	0.34 ± 0.02
E	0.5	0.59 ± 0.04	0.34 ± 0.01
